# *APOC3* genetic variation, serum triglycerides, and risk of coronary artery disease in Asian Indians, Europeans, and other ethnic groups

**DOI:** 10.1186/s12944-021-01531-8

**Published:** 2021-09-21

**Authors:** Shiwali Goyal, Yosuke Tanigawa, Weihua Zhang, Jin-Fang Chai, Marcio Almeida, Xueling Sim, Megan Lerner, Juliane Chainakul, Jonathan Garcia Ramiu, Chanel Seraphin, Blair Apple, April Vaughan, James Muniu, Juan Peralta, Donna M. Lehman, Sarju Ralhan, Gurpreet S. Wander, Jai Rup Singh, Narinder K. Mehra, Evgeny Sidorov, Marvin D. Peyton, Piers R. Blackett, Joanne E. Curran, E. Shyong Tai, Rob van Dam, Ching-Yu Cheng, Ravindranath Duggirala, John Blangero, John C. Chambers, Charumathi Sabanayagam, Jaspal S. Kooner, Manuel A. Rivas, Christopher E. Aston, Dharambir K. Sanghera

**Affiliations:** 1 Department of Pediatrics, College of Medicine, University of Oklahoma Health Sciences Center, 940 Stanton L. Young Blvd., Rm 317 BMSB, Oklahoma City, OK 73104 USA; 2 Department of Biomedical Data Science, School of Medicine, Stanford University, Stanford, California USA; 3 Department of Epidemiology and Biostatistics, Imperial College London, London, W2 1PG UK; 4 Department of Cardiology, Ealing Hospital, Middlesex, UB1 3HW UK; 5 Saw Swee Hock School of Public Health, National University of Singapore and National University Health System, Singapore , 117549 Singapore; 6 Department of Human Genetics and South Texas Diabetes and Obesity Institute, University of Texas Rio Grande Valley, Brownsville, TX USA; 7 Department of Surgery, Oklahoma University of Health Sciences Center, Oklahoma City, OK USA; 8 Department of Neurology, University of Oklahoma Health Sciences Center, 920 S. L Young Blvd #2040, Oklahoma City, OK 73104 USA; 9 Departments of Medicine and Epidemiology and Biostatistics, University of Texas Health San Antonio, San Antonio, TX USA; 10 Hero DMC Heart Institute, Ludhiana, Punjab India; 11 Central University of Punjab, Bathinda, Punjab India; 12 All India Institute of Medical Sciences and Research, New Delhi, India; 13 Department of Pediatrics, Section of Endocrinology, Oklahoma University of Health Sciences Center, Oklahoma City, OK USA; 14 Department of Medicine, Yong Loo Lin School of Medicine, National University Health System, Singapore , 119228 Singapore; 15 Department of Nutrition, Harvard T.H. Chan School of Public Health, Boston, MA USA; 16 Duke-NUS Medical School, Singapore, 169857 Singapore; 17 Singapore Eye Research Institute, Singapore National Eye Centre, Singapore, 168751 Singapore; 18 National University of Singapore, Singapore, 119077 Singapore; 19 Lee Kong Chan School of Medicine, Nanyang Technological University, Singapore, 308232 Singapore; 20 Imperial College Healthcare NHS Trust, Imperial College London, London, W12 0HS UK; 21 MRC-PHE Centre for Environment and Health, Imperial College London, London, W2 1PG UK; 22 National Heart and Lung Institute, Imperial College London, London, W12 0NN UK; 23 Department of Pharmaceutical Sciences, University of Oklahoma Health Sciences Center, Oklahoma City, OK USA; 24 Department of Physiology, College of Medicine, University of Oklahoma Health Sciences Center, Oklahoma City, OK USA; 25 Oklahoma Center for Neuroscience, University of Oklahoma Health Sciences Center, Oklahoma City, OK USA; 26 Harold Hamm Diabetes Center, University of Oklahoma Health Sciences Center, Oklahoma City, OK USA

**Keywords:** ApoC-III, Rare and common variants, Mendelian randomization, Triglyceride, Coronary artery disease risk, Asian Indians

## Abstract

**Background:**

Hypertriglyceridemia has emerged as a critical coronary artery disease (CAD) risk factor. Rare loss-of-function (LoF) variants in apolipoprotein C-III have been reported to reduce triglycerides (TG) and are cardioprotective in American Indians and Europeans. However, there is a lack of data in other Europeans and non-Europeans. Also, whether genetically increased plasma TG due to ApoC-III is causally associated with increased CAD risk is still unclear and inconsistent. The objectives of this study were to verify the cardioprotective role of earlier reported six LoF variants of *APOC3* in South Asians and other multi-ethnic cohorts and to evaluate the causal association of TG raising common variants for increasing CAD risk.

**Methods:**

We performed gene-centric and Mendelian randomization analyses and evaluated the role of genetic variation encompassing *APOC3* for affecting circulating TG and the risk for developing CAD.

**Results:**

One rare LoF variant (rs138326449) with a 37% reduction in TG was associated with lowered risk for CAD in Europeans (*p* = 0.007), but we could not confirm this association in Asian Indians (*p* = 0.641). Our data could not validate the cardioprotective role of other five LoF variants analysed. A common variant rs5128 in the *APOC3* was strongly associated with elevated TG levels showing a *p*-value 2.8 × 10^− 424^. Measures of plasma ApoC-III in a small subset of Sikhs revealed a 37% increase in ApoC-III concentrations among homozygous mutant carriers than the wild-type carriers of rs5128. A genetically instrumented per 1SD increment of plasma TG level of 15 mg/dL would cause a mild increase (3%) in the risk for CAD (*p* = 0.042).

**Conclusions:**

Our results highlight the challenges of inclusion of rare variant information in clinical risk assessment and the generalizability of implementation of ApoC-III inhibition for treating atherosclerotic disease. More studies would be needed to confirm whether genetically raised TG and ApoC-III concentrations would increase CAD risk.

**Supplementary Information:**

The online version contains supplementary material available at 10.1186/s12944-021-01531-8

## Highlights

- There is a strong influence of genetic factors for controlling circulating triglycerides (TG). However, the causal association between hypertriglyceridemia and the development of coronary artery disease (CAD) is unclear. Earlier published studies have primarily examined individuals from European populations. For the first time, in this study, we have included data from Asian Indians with Europeans and other ethnic groups to identify the causal association of genetically raised TG due to ApoC-III with the risk for CAD.
- We also evaluated the role of six earlier reported loss-of-function rare variants in *APOC3* for protecting CAD. Only one variant IVS2 + 1G-A (rs138326449), showed ~ 37% reduction in TG and had a significantly lower risk for CAD (0.64 95%CI 0.47–0.88; *p* = 0.007), but its phenotypic effects could not be confirmed in Asian Indians.
- A common variant rs5128 in the *APOC3* was strongly associated with elevated TG across all cohorts showing a meta-analysis *p*-value of 2.80 × 10^− 424^.
- We have used genetic instrumental variable methods to obtain estimates of the causal association between circulating TG levels and CAD for determining the direction of causality by performing a Mendelian randomization study. Genetically instrumented per 1SD increment of plasma TG level of 15 mg/dL would cause a mild increase of 3% in the risk for CAD (*p* = 0.042).

## Introduction

Hypertriglyceridemia (HTG) is a common disorder of blood lipids associated with elevated blood triglyceride (TG) concentration. Except for the very rare monogenic form of HTG [1], the common form of HTG is also a heritable genetic condition and a risk factor for coronary artery disease (CAD). Genetic variation in several genes involved in TG hydrolysis, obesity, and metabolic syndrome, are implicated in influencing polygenic HTG, which makes the clinical management of HTG extremely challenging [2]. HTG can also develop from secondary causes such as poorly managed type 1 or type 2 diabetes, hypothyroidism, renal insufficiency, and as side effects of certain medications (estrogen, beta-blockers, diuretics, glucocorticoids, antidepressants, and antipsychotics) [3, 4]. Lipoprotein lipase (LPL) is an important regulator of lipid metabolism and plays a key role in the hydrolysis of TG-rich very-low-density lipoproteins (VLDL), intermediate-density lipoproteins, and chylomicron remnants [5]. Apolipoprotein C-III (ApoC-III) is an inhibitor of LPL and obstructs the hepatic uptake of TG-rich lipoproteins and lipoprotein remnants. A blood TG level < 150 mg/dL is considered normal. An increase of TG > 150 mg/dL (150–199 mg/dL) is classified as moderate HTG, and > 200 mg/dL (200 or 250 mg/dL) is considered high HTG, and above 500 mg/dL is defined as severely elevated HTG [6–8]. Elevated blood ApoC-III is correlated with increased TG and increased risk for myocardial infarction and CAD [9]. Average blood ApoC-III concentration of ~ 10 mg/dL or lower correlates with normal TG and > 20 mg/dL with HTG [10]. However, very few investigators have measured ApoC-III levels in human studies.

The *APOC3* gene that encodes ApoC-III is located within the *APOA5-APOA4-APOC3-APOA1* gene cluster in the chromosomal region 11q23.3. Common genetic variants-specifically in the promoter or 5′ (T-455C, C-482 T) and 3′ UTR (*Sst-1* or rs5128) regions, have consistently been shown to be robustly associated with blood lipid levels in candidate gene- and genome-wide association studies (GWAS) [11, 12]. Meta-analyses of GWAS in multi-ethnic populations have shown strong association signals between the *APOA5-APOA4-APOC3-APOA1* gene cluster and circulating TG levels [13, 14]. However, whether the genetically enhanced TG (owing to the *APOC3* common variants) increases CAD risk remains inconclusive across many studies.

Resequencing studies of the *APOC3* gene have identified rare null variants with strong phenotypic effects associated with lower TG levels in recent studies [15, 16]. A rare missense variant (A43T; rs147210663; earlier notation A23T) in the *APOC3* found in Yucatan Indians was linked with ApoC-III deficiency with reduced blood TG concentration [17]. Another rare loss-of-function (LoF) variant in *APOC3* (initially found in an Amish population) (R19X; rs76353203) was associated with reduced TG levels. The heterozygous minor allele carriers had a 50% reduced ApoC-III concentration compared to non-carriers [15]. A sequencing study on the Danish population from Europe and the results of the Exome Sequencing Project of the National Heart, Lung, and Blood Institute on epidemiological cohorts (predominantly of US white) also confirmed earlier known rare variants to be associated with reduced TG and, hence, protective against CAD [16, 18]. However, there is a paucity of data on whether these or other rare and common variants in the *APOC3* would show the same phenotypic effects that would be cardio-protective in other European and non-European populations, particularly the populations of Asian Indians. People of the Indian diaspora contribute the highest number of CAD deaths worldwide and in their countries of origin [19]. Epidemiological studies suggest that people of Asian Indian descent exhibit an increased predisposition for earlier and acute myocardial infarction and atherosclerotic cardiovascular disease that is not explained by traditional risk factors [20]. Therefore, the purpose of this study was: 1) to perform gene-centric analysis of the *APOC3* region to identify common and rare variants associated with TG; 2) to verify the cardioprotective role of earlier reported LoF/reduced-function variants of the *APOC3*; and 3) to analyze whether genetically raised TG due to *APOC3* variation would increase the development of CAD, in Asian Indian and other multi-ethnic cohorts comprising 396,644 individuals.

## Methods

### Study cohorts

Our study investigated 396,644 individuals comprised of 4659 Asian Indians from India and the US (AIDHS/SDS) [21–23]; 11,339 Asian Indians from London, UK (LOLIPOP) [24]; 2713 Asian Indians (MEC_Indian, and SINDI) from Singapore [25], 7885 Asian Indians from UK BIOBANK (UKBB) [26], and 2999 Chinese (MEC_Chinese and DC_SP2) from Singapore [27]. Additionally, we included 2153 Europeans from London, UK (LOLIPOP) [24]; 362,043 Europeans from UKBB [28]; 2341 Mexican Americans from San Antonio (SAMAFS) [29], and 512 multi-ethnic individuals from Oklahoma (MISS_OLIVER) [30]. Details of clinical and demographic characteristics of all cohorts are described in Table 1. Diagnostic criteria for CAD cases and non-CAD controls in participating study cohorts are summarized in Supplementary Table 1.

**Table 1:** Clinical attributes of study cohorts

Cohort	AIDHS/SDS ***N*** = 4659	LOLIPOP_AI ***N*** = 11,339	LOLIPOP_EU ***N*** = 2153	SINGAPORE_AI ***N*** = 2713	SINGAPORE_CHS ***N*** = 2999	SAMAFS (MEXICANS) ***N*** = 2341	MISS_OLIVER (MULTIETHNIC) ***N*** = 512	UKBB_AI ***N*** = 7885	UKBB_EU ***N*** = 362,043
**Ancestry**	AI	AI	EU	AI	CHS	MEX	MULTI-ETHNIC	AI	EU
**N**	4659	11,339	2153	2713	2999	2341	512	7885	362,043
**Female (%)**	44	19	15	51	49	58	52	45	54
**CAD (%)**	17	26	27	NA	NA	10	27	7	4
**Age (yrs)**	52.14 ± 13.21	52.13 ± 9.67	56.99 ± 8.96	55.08 ± 9.24	54.49 ± 8.59	53.78 ± 15.12	53.78 ± 16.47	66.68 ± 8.3	70.29 ± 7.39
**BMI (kg/m**^**2**^**)**	26.78 ± 4.84	27.91 ± 4.34	29.62 ± 5.18	26.66 ± 5.06	24.32 ± 3.81	32.31 ± 7.7	29.26 ± 6.52	26.91 ± 7.42	29.41 ± 5.61
**TG (mg/dL)**	169.94 ± 107.59	159.24 ± 100.9	176.79 ± 131.9	154.53 ± 94.58	130.68 ± 79.32	165.33 ± 105.36	147.92 ± 97.23	180.73 ± 104.43	175.41 ± 99.51
**HDL-C (mg/dL)**	40.81 ± 14.47	48.43 ± 12.04	48.34 ± 10.14	41.93 ± 12.09	54.10 ± 13.87	47.98 ± 13.98	41.46 ± 12.98	46 ± 11.52	51.01 ± 13.35
**LDL-C (mg/dL)**	110.40 ± 38.42	122.33 ± 34.97	117.43 ± 35.87	125.99 ± 34.74	117.27 ± 30.86	113.3 ± 32.36	109.67 ± 39.96	117.34 ± 30.93	122.82 ± 32.94

Values are displayed in mean ± SD; *AI* Asian Indians, *EU* Europeans, *CHS* Chinese, *MEX* Mexicans, *CAD* Coronary Artery Disease, *BMI* Body-Mass Index, *TG* Triglycerides, *HDL-C* High-Density Lipoprotein-Cholesterol, *LDL-C* Low-Density Lipoprotein-Cholesterol, *AIDHS/SDS* Asian Indian Diabetic Heart Study/Sikh Diabetes Study, *LOLIPOP* The London Life Sciences Prospective Population Study, *SAMAFS* San Antonio Mexican American Family Studies, *UKBB* UK BIOBANK, *MISS-OLIVER* Metabolome in Ischemic Stroke Study and Oklahoma Multiethnic CardioVascular Health Disparity Study

### Genotyping and sequencing

Details of genome-wide genotype and sequencing information are described in the online Supplementary Section and Supplementary Table 2.

### Serum/plasma ApoC-III measurements using ELISA

Circulating concentrations of ApoC-III were quantified using frozen serum aliquots by enzyme-linked immunosorbent assay (ELISA) kits from Thermofisher Scientific (Waltham, MA, USA) following the manufacturer’s instructions. Briefly, the Thermofisher’s Human ApoC-III ELISA Kit was based on solid-phase sandwich ELISA technology with a target-specific antibody pre-coated onto 96-well plates, and test samples were added to the wells in duplicates. A biotinylated detection polyclonal antibody was added subsequently and then followed by washing with 1x PBS buffer. Avidin-biotin-peroxidase complex was added, and unbound conjugates were washed away with 1x PBS buffer. Horseradish peroxidase (HRP) substrate 3,3′,5,5′-tetramethylbenzidine (TMB) was used to visualize HRP enzymatic reaction. TMB was catalyzed by HRP to produce a blue color product that changed into yellow after adding an acidic stop solution. The density of yellow color is proportional to the Human ApoC-III concentration of sample captured in the plate, and the optical density (OD) absorbance was measured at 450 nm by GENEMATE microplate reader. Samples were blinded for ApoC-III measurements, and each specimen was run in duplicate. A standard curve was generated and used to determine the ApoC-III level in the tested serum samples. The ApoC-III measures were performed in a selected subset of 38 Sikh individuals, half with wild-type (CC), half with mutant genotype (GG).

### Statistical analysis

#### Genetic association analysis

Each study site provided summary statistics of the association for all available rare variants (with a minor allele frequency (MAF) < 1%) and common variants (MAF > 5%) within the region encompassing the open reading frame of the *APOC3*. Statistical evaluations of genetic effects of common and rare variants on lipid panel and other metabolic traits and CAD were performed using multivariable logistic and linear regression models using covariates such as age, gender, BMI, and diabetes. Principal components (PC) were included in the models to adjust for the population structure as described previously [21, 22]. As the existing HapMap and 1000 Genomes data do not include Sikhs, the 5 principal components used for this correction were estimated using our Sikh population samples as described earlier [21]. We genotyped the discovery and the replication datasets of AIDHS/SDS set using Human 660W Quad BeadChip and Illumina’s Global Screening Array (Illumina, Inc., San Diego, CA) as described previously [23]. We also performed pairwise identity-by-state (IBS) clustering in PLINK across all individuals to assess population substructure due to cryptic relatedness and to remove outliers. Samples with < 93% call rate, SNPs with < 95% call rate, SNPs with deviation from Hardy-Weinberg Equilibrium (HWE; *p* < 10^− 6^), and individuals with gender discrepancies were excluded from the analysis. To increase genome coverage, imputation was performed using Minimac4 [31] (https://imputationserver.sph.umich.edu/) with 1000G Phase 3 v5 multi-ethnic reference panel in NCBI Build 37 (hg19) coordinates as described [22, 23]. Quality control for the imputed SNPs included removal of variants with an imputation certainty info score (R^2^) < 0.8 and SNPs significantly deviated from HWE in controls (*p* < 1 × 10^− 6^). From a total of 46,511,137 variants, 9,400,020 variants with MAF > 1% were available after the quality control. For this study, we performed a locus-wide association of chromosome 11 encompassing *APOC3* and its flanking regions (116698024–116,710,387) for common variant association analysis. We selected all independent signals (*p* < 10^− 3^) from Sikh discovery (*n* = 820) with MAF > 5% for replication and look-up in GWAS in replication cohorts of Sikhs (AIDHS/SDS) (*n* = 3839) and other Asian Indians (from Asian Indian component of the LOLIPOP, Singapore, and South Asian component of the UKBB), Europeans (LOLIPOP, UKBB), and Others (including Singapore Chinese [MEC_CHS], Mexican American [SAMAFS], and African American, Hispanic and mixed ethnicity [MISS-OLIVER]) (Table 1). General mixed linear models were used to test the impact of genetic variants on transformed continuous traits using the variance-component test adjusted for the random-effects of relatedness to account for family structure and fixed effects of age, gender, BMI, and diabetes status and PCs assuming an additive genetic model using SVS version 8.8.3 (Golden Helix, Bozeman, MT, USA) [23]. Continuous traits with skewed sampling distributions (e.g., TG) were log-transformed before statistical analysis. However, for illustrative purposes, values were re-transformed into the original measurement scale. Meta-analysis was performed on selected SNPs showing significant association in most of the study cohorts. To combine common SNP association results from all cohorts, both fixed and random effect inverse variance metanalysis implemented in METAL [32] was employed. Two-tailed *p*-values lower than 5 × 10^− 8^ were considered genome-wide significant. The LocusZoom standalone tool was used to generate a regional association plot for the *APOC3* locus [33]. The Forest Plot Viewer (http://ntp.niehs.nih.gov/ntp/ohat/forestplot/) and PRISM (https://www.graphpad.com/scientific-software/prism/) were used to generate forest plots. For determining the association of the five known rare variants for CAD, a value of 0.5 is added to all the cells where zeros caused problems in the computation of odds ratios as described [34].

#### Mendelian randomization (MR) studies

In general, elevated plasma TG is known to increase the risk for CAD in epidemiological and prospective studies; however, whether the genetically increased TG is causally related to the development of CAD is still unknown. The MR approach uses the genetic variation as an instrumental variable (IV) to define the causal association between a risk factor or exposure (e.g., TG) with the disease (e.g., CAD) [35, 36]. The basic principle of the MR methodology is based on the assumptions that the IV (*APOC3* genotype) associated with the phenotype (TG), is independent of known and unknown confounders, and it can only influence the outcome (i.e., CAD) through the exposure (TG). To ensure that the MR assumptions were not violated, we chose the *APOC3* variant with the strongest independent association with the exposure (i.e., TG) across all cohorts and pruned the SNPs in linkage disequilibrium (LD). Further, the SNP used as a Mendelian instrument was not associated with the outcome via exposure to other confounding factors (BMI, age, gender, and diabetes). For assessing the causal association of exposure with the outcome, we used a one-sample MR using the inverse-variance method.

## Results

### Genetic associations of common variants with TG and CAD

**Fig. 1:**
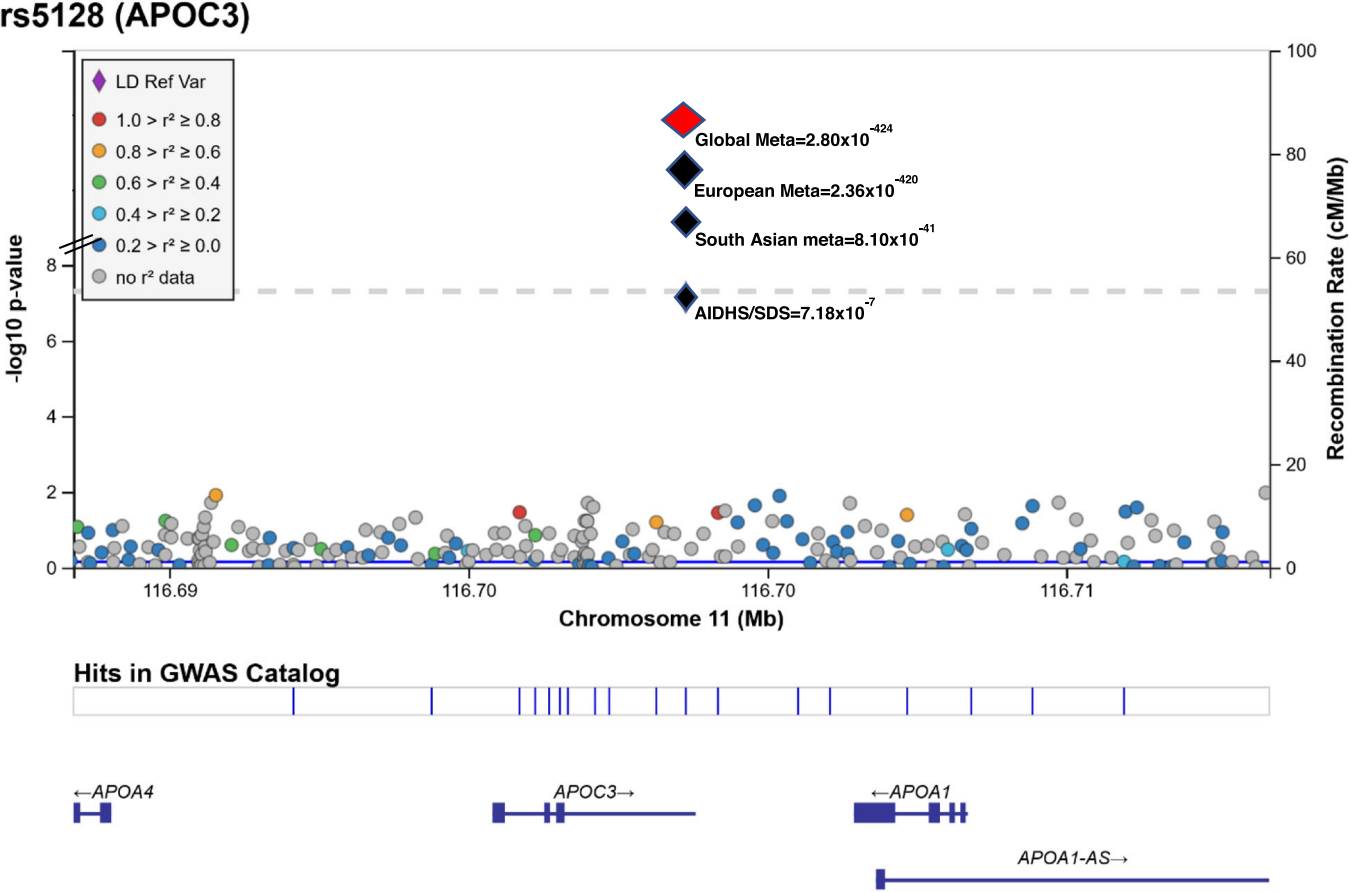
Regional association plot for the *APOC3* common variant rs5128 showing association with plasma triglyceride levels. SNPs from the *APOC3* region are plotted by position on the x-axis versus association with triglyceride levels (−log10 P) on the y-axis. The black diamond signifies p-values of the studied SNP (rs5128) in Sikhs and in the combined analysis using replication studies. Global meta-analysis results are depicted by a red diamond at the top of the plot. The SNPs surrounding the most significant SNP are color-coded to reflect their LD with this SNP. We present linkage disequilibrium (LD) using the GIH panel (Gujarati Indians in Houston), the closest HapMap population to the Sikhs. At the bottom of the plot, the locations of known genes in the region are shown

**Fig. 2:**
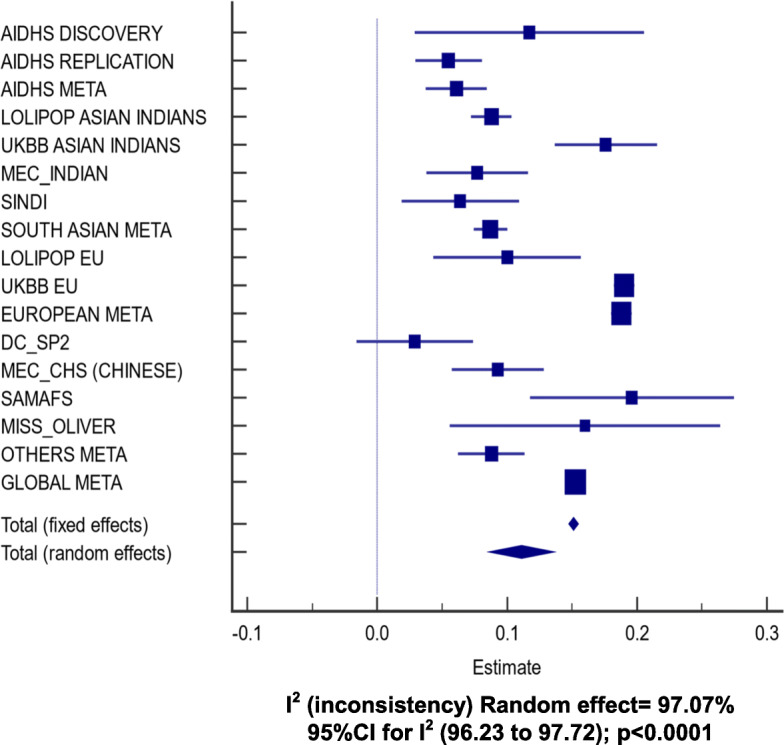
Random-effect metanalysis showing allelic effects of the *APOC3* common variant rs5128 with plasma TG levels among 396,644 participants from different ethnic cohorts. Analysis was adjusted for age, gender, BMI, diabetes, and 5 principal components using linear regression

**Fig. 3:**
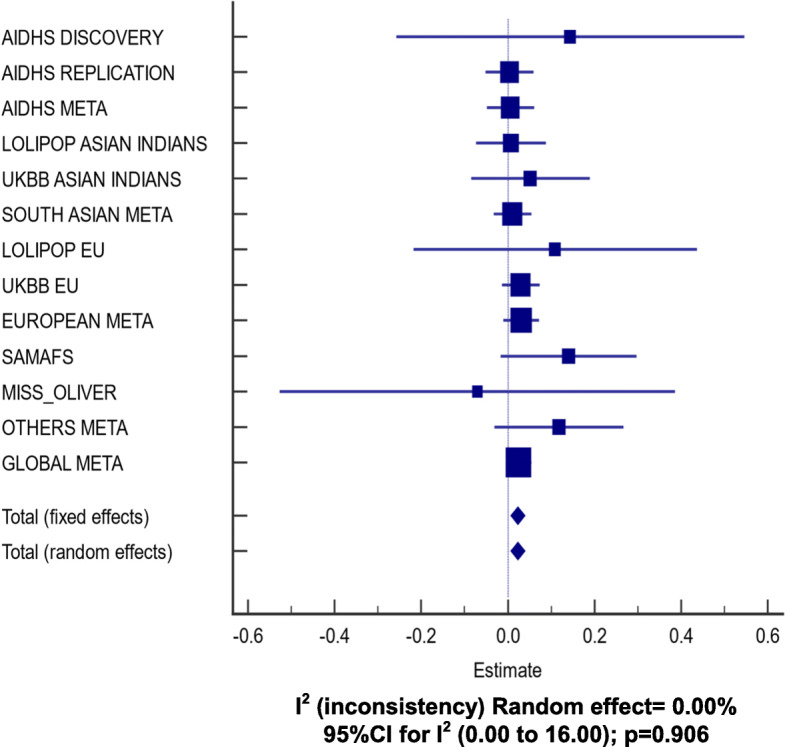
Random-effect metanalysis showing allelic effects of the *APOC3* common variant rs5128 with CAD among 390,932 participants from different ethnic cohorts. Analysis was adjusted for age, gender, BMI, diabetes, and 5 principal components using logistic regression

A gene-centric association analysis was performed on all directly genotyped and high-quality imputed SNPs within the *APOC3* region with MAF ≥5% to identify gene variants associated with TG concentrations using multiple linear regression controlling for age, gender, BMI, diabetes, and PCs. Of all common variants surveyed from the open reading frame of the *APOC3*, two variants [rs5128 (3′ UTR) (Fig. 1) and rs734104 (intronic)] showed a strong association with increased plasma TG in Sikhs (AIDHS). These two variants were in LD with each other (*r*^*2*^ = 0.78), therefore, we only used one variant (rs5128) as a genetic instrument (IV) for MR studies. The estimates (β ± SE) for rs5128 for effecting TG levels was 0.06 ± 0.01; *p* = 7.18 × 10^− 7^ after adjusting for the effects of age, gender, BMI, diabetes, and PCs. (Fig. 2, Supplementary Table 3). Similarly, rs5128 showed a robust replication with significantly raised TG (0.09 ± 0.01; *p* = 8.10 × 10^− 41^) in other independent Asian Indian cohorts (LOLIPOP, UKBB_AI, MEC_INDIAN, and SINDI). Apart from South Asians, the highly significant association of this variant was also observed in Europeans (LOLIPOP, and UKBB) (0.19 ± 0.004; *p* = 2.36 × 10^− 420^) and others (MEC_CHS, DC_SP2, SAMAFS, MISS_OLIVER) (0.09 ± 0.01; *p* = 1.15 × 10^− 11^), adjusting for the effects of age, gender, BMI, diabetes, and PCs. A global meta-analysis across all cohorts showed strong allelic results of rs5128 for increasing plasma TG (0.15 ± 0.004, *p* = 2.80 × 10^− 424^ (Fig. 2; Supplementary Table 3). No association of this variant was observed with T2D, LDL-cholesterol, and total cholesterol (data not shown). A per SD increase of TG of 15 mg/dL ± 0.004 mg/dL (*p* = 2.80 × 10^− 424^) for rs5128 is predicted to increase CAD risk by 3% 95% CI [0–5%]; *p* = 0.042. (Fig. 3; Supplementary Table 3).

**Fig. 4:**
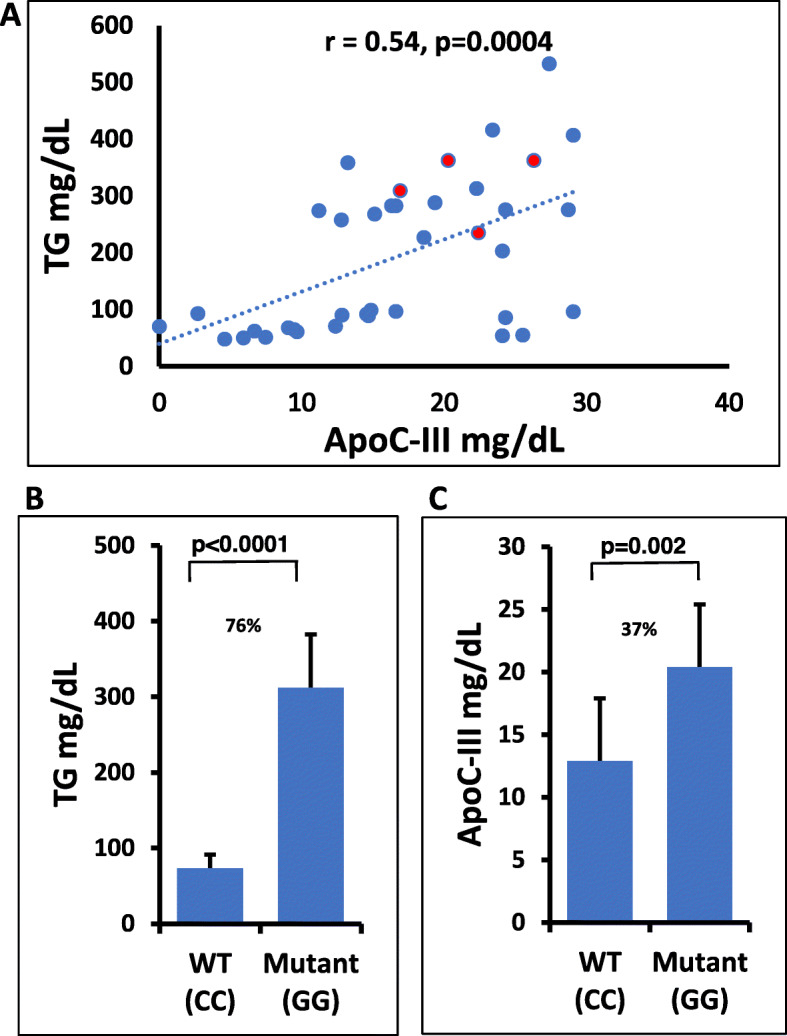
Bar graphs show mean differences of (**A**) Scatter plot of ApoC-III concentration (mg/dL) X-axis vs TG levels (mg/dL) Y-axis in the homozygous mutant (GG) and wild-type (CC) genotype carriers of Sikh ancestry. Red dots indicate individuals with CAD (**B**) plasma TG mg/dL and (**C**) ApoC-III concentrations (mg/dL) among mutant (GG) and wild-type genotype carriers (CC) of rs5128 in Sikhs

We also explored if genetically raised TG will correlate with increased ApoC-III concentration. For this, we measure serum ApoC-III levels in a small subset of 38 Sikh individuals, half with homozygous wild type (CC) and half with mutant (GG) alleles of rs5128. The correlation between serum TG and serum ApoC-III levels was significantly positive (*r* = 0.54, *p* = 0.0004) (Fig. 4A) which is very similar to an earlier published study (*r* = 0.59; *p* < 1 × 10^− 4^) [37]. While there was a 76% increase in TG level among the mutant homozygous carriers than the wild type carriers (Fig. 4B), the ApoC-III levels increased 37% among the mutant homozygous carriers compared to the wild type carriers (Fig. 4C).

### Association of previously known *APOC3* rare variants with TG and CAD

**Fig. 5:**
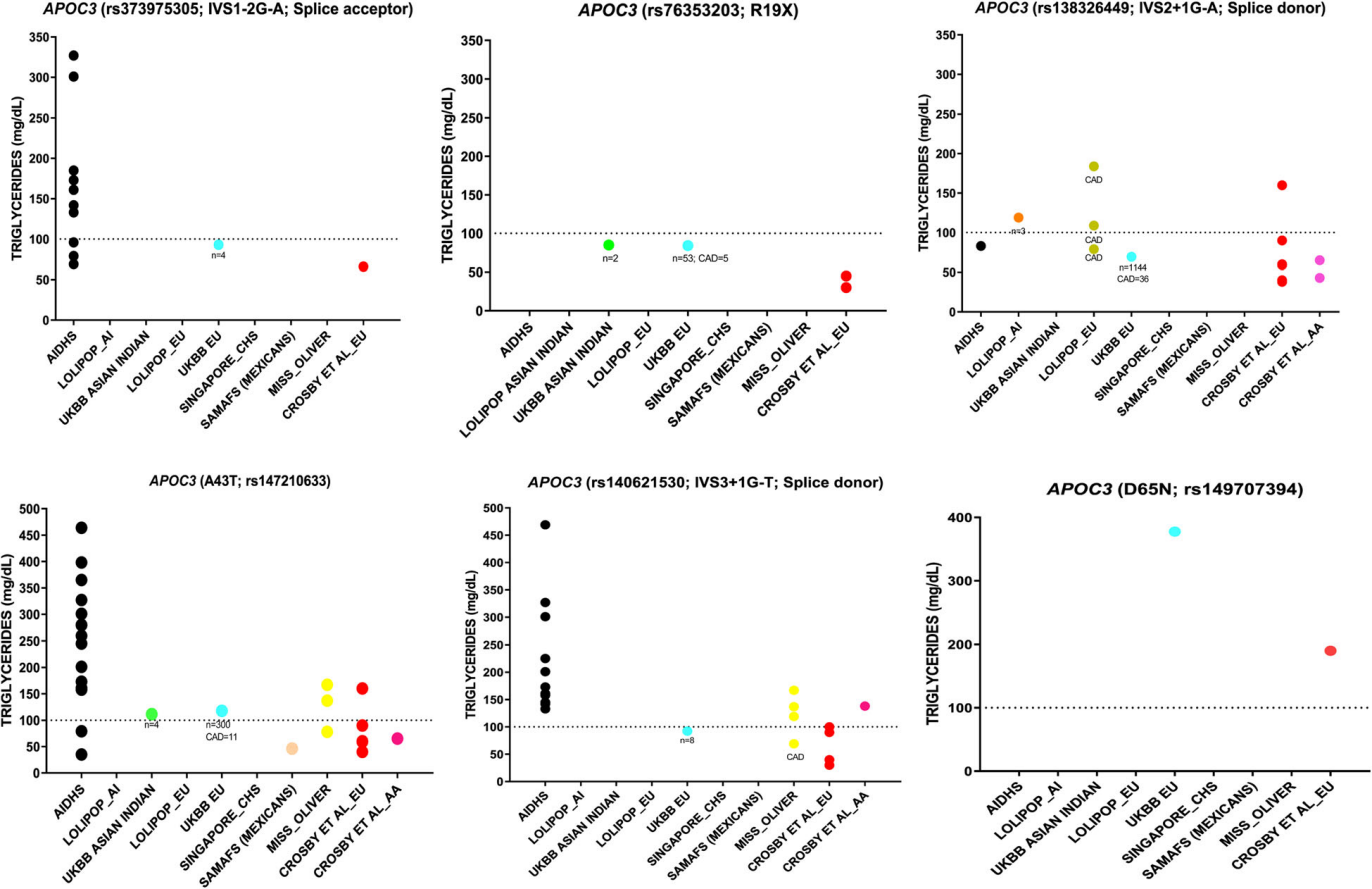
Distribution of earlier known rare functional variants in *APOC3* and their relation with plasma TG. Figures present the study-wise distribution of earlier reported rare variants (x-axis) and corresponding TG levels (y-axis). Each dot in the graph represents the carrier(s) of the variant. A cut-off of 100 mg/dL was used to define low or normal plasma TG levels. Variants with a large number of carriers from the UKBB, only mean levels were available for plasma TG

We evaluated the role of previously reported rare variants for their association with TG and CAD in our cohort. We observed a total of 56 carriers of null variant rs76353203 (R19X): one carrier of Sikh ancestry (India), two Asian Indians, and 53 European carriers from UKBB (Table 2). Five (9%) of the 53 European carriers of this variant from UK BIOBANK were affected with CAD (Fig. 5).

**Table 2:** Allelic distribution of previously known *APOC3* rare variants and their association with TG, HDL-C, and CAD

Variant	Carrier	Non-Carrier	% Carriers	Mean TG (mg/dL) Carrier/Non-carrier	Mean HDL-C (mg/dL) Carrier/Non-carrier	CAD Among Carriers	CAD Among Non-carriers	Odds Ratio/CI/P (CAD)
**rs373975305 (IVS1-2G-A)**	16	390,916	0.004	129.8/164.2 (−21%)	46.5/45.4 (2.4%)	0/16	20,145/370771	0.56 95%CI (0.03–9.30); *p* = 0.684
**rs76353203 (R19X)**	56	390,876	0.014	84.5/163.4 (−48.3%)	60.5/45.7 (32.4%)	5/51 (8.9%)	20,156/370720	1.80 95%CI (0.72–4.52); *p* = 0.208
**rs138326449 (IVS2 + 1G-A)**	1157	389,775	0.297	98.9/157.9 (−37.4%)	61.4/47.1 (30.4%)	39/1118 (3.5%)	20,105/369670	**0.64 95%CI (0.47–0.88);*p*** **= 0.007**
**rs147210663 (A43T)**	326	390,606	0.083	122.5/163.4 (− 25%)	50.4/45.7 (10.3%)	11/315 (3.5%)	20,131/370475	0.64 95%CI (0.35–1.17); *p* = 0.150
**rs140621530 (IVS3 + 1G-T)**	24	390,908	0.006	133.1/164.2 (−18.9%)	52.9/45.4 (16.5%)	1/23 (4%)	30,010/360898	0.52 95%CI (0.07–3.87); *p* = 0.526

Variant data from Singapore Chinese (MEC_ Chinese) and Singapore Asian Indians (MEC_ Indian and SINDI) were not included because of the lack of information on CAD

All other known variants detected in AIDHS/SDS and other cohorts were not associated with low TG levels as seen in white populations in published studies. For instance, 12 heterozygous carriers (IVS3 + 1GT; rs140621530) of Punjabi Sikh ancestry (AIDHS/SDS) had high TG (ranging from 133 to 469 mg/dL); three heterozygous US white carriers from MISS_OLIVER also had high TG levels (average 141 mg/dL). In contrast, one African American carrier from MISS_OLIVER had low TG (78 mg/dL), but this individual was a CAD patient. In the UKBB European cohort, eight carriers were observed with a mean TG level of 92.2 mg/dL. A single carrier of a known rare variant D65N (rs149707394) observed in UKBB (European) had a plasma TG of 377.7 mg/dL (Fig. 5).

Our data detected 18 carriers of A43T (rs147210663) in AIDHS/SDS and only two of 18 showed reduced TG (57 ± 31 mg/dL; range 35–79 mg/dL) while the remaining 16 individuals had elevated TG (mean 267 ± 101 mg/dL; range 142 to 464 mg/dL). One of the two carriers of A43T with very low TG (35 mg/dL) also had low HDL-C (22 mg/dL). Four carriers of A43T were also observed in UKBB (Asian Indian) with a mean TG level of 111.3 mg/dL. Furthermore, 300 carriers of A43T of UKBB (European) had a mean TG of 117.7 mg/dL, and 11/300 (4%) had CAD. A single Mexican American carrier from SAMAFS had a low TG level of 46 mg/dL and an African American from MISS_OLIVER had low TG (69 mg/dL) (Fig. 5).

We performed a combined association analysis of five known rare variants (found in most of the cohorts). Even though the plasma mean TG levels were significantly lower among the variant allele carriers vs. the wild type carriers, none of these variants exhibited any significant protection against CAD except for the rs138326449 (IVS2 + 1G-A). As shown in Table 2, the splice variant rs138326449 was associated with a significantly reduced risk for CAD (OR 0.64 95%CI 0.47–0.88; *p* = 0.007) with ~ 37% reduction in TG in the combined sample. However, the cardioprotective effect of this variant was not significant in Asian Indians when data was analyzed separately (OR = 0.50 95%CI 0.03–9.26; *p* = 0.641) (Table 3). Similarly, no other variant revealed any significant protective association against CAD despite showing a reduced concentration of TG in Europeans or other populations used in our study (Tables 2 and 3).

**Table 3:** Allelic distribution of previously known *APOC3* rare variants and their association with TG, HDL-C, and CAD only in Asian Indians

Variant	Carrier	Non-Carrier	% Carriers	Mean TG (mg/dL) Carrier/Non-carrier	Mean HDL mg/dL Carrier/Non-carriers	CAD Among Carriers	CAD Among Non-carriers	Odds Ratio/CI/P (CAD)
**rs373975305 (IVS1-2G-A)**	12	23,871	0.05	166.6/173.6 (−4%)	44/42.6 (3.3%)	0/12	4364/19507	0.18 95%CI (0.01–3.02); *p* = 0.233
**rs76353203 (R19X)**	3	23,880	0.01	84.9/171.5 (−50.5%)	55.1/43.3 (27.3%)	0/3	4364/19516	0.64 95%CI (0.03–12.37); *p* = 0.767
**rs138326449 (IVS2 + 1G-A)**	4	23,879	0.02	101/165.8 (−39.1%)	46.7/46.2 (1.1%)	0/4	4351/19528	0.50 95%CI (0.03–9.26); *p* = 0.641
**rs147210663 (A43T)**	22	23,861	0.09	175.2/171.5 (2.2%)	42.7/43.4 (−1.6%)	0/22	4364/19497	0.09 95%CI (0.01–1.64); *p* = 0.106
**rs140621530 (IVS3 + 1G-T)**	12	23,871	0.05	221/173.6 (27.3%)	43/42.6 (0.9%)	0/12	4364/19507	0.18 95%CI (0.01–3.02); *p* = 0.233

Variant data from Singapore Asian Indians (MEC_ Indian and SINDI) were not included because of the lack of information on CAD

## Discussion

The role of ApoC-III in lipid metabolism has been well-documented in human and animal studies. However, the molecular mechanism of the action of ApoC-III in lipid metabolism and CAD is still unclear. The discovery of LoF or reduced function variants in *APOC3* and other lipid genes has started shedding light on their putative molecular role in lipid metabolisms. Because these LoF/reduced function variants were shown to be cardioprotective in recent studies, there has been a consensus to therapeutically inhibit ApoC-III for treating dyslipidemia and prevent CAD susceptibility using antisense oligos [38]. However, as most of the large-size studies have been heavily focused on Europeans, whether the therapeutic inhibition of ApoC-III (based on the LoF variants) would be universally effective to prevent CAD in all dyslipidemic patients has not been explored. Moreover, in this study, we, for the first time, are reporting the role of *APOC3* genetic variation on TG and CAD in Asian Indians, who are grossly underrepresented in genetic, clinical, biomarker research despite having a huge burden of cardiometabolic conditions. Also, because of the rapidly expanding role of genetic testing in disease prediction or diagnosis, the transferability of these findings in other diverse ethnic groups is imperative. Here, we are reporting the role of *APOC3* rare variants (known and novel) and common variants for their effects on TG, and CAD in Asian Indians, Mexicans, Chinese, and Europeans from India, Singapore, the UK, and the USA.

The MR analysis showed a TG raising common variant (rs5128) in the *APOC3* gene may be involved in a modest increase in CAD risk by elevating circulating TG levels. A genetically instrumented per 1-SD increase in TG of 15 mg/dL would modestly increase CAD risk to 3% 95%CI (0–5%; *p* = 0.042) (Fig. 3). Agreeing with earlier reports, this variant was not associated with LDL-cholesterol in any of the participating cohorts in this study [39]. These results suggest that the observed association of *APOC3* with CAD could be in the pathway independent of cholesterol metabolism. The two *APOC3* SNPs, rs5128 and rs734104, were independently associated with increased TG concentrations. Notably, except for these two variants, no other SNP within the open reading frame of the *APOC3* was associated with TG levels in any studied cohorts. The rs5128, also known as *Sst-I*, resides in the 3′-untranslated region of the *APOC3* gene and might enable microRNA binding and hence can change the transcriptional activity of ApoC-III, which ultimately would lead to higher plasma ApoC-III levels, increased TG, and the increased risk for CAD [40].

Further, we examined the role of earlier published LoF or reduced function splice/missense rare variants known to be protective against the CAD. One splice-donor variant IVS2 + 1G-A (rs138326449), which predominantly segregated in populations of European ancestry (with 99.6% European heterozygous carriers), showed an ~ 37% reduction in TG and had a significantly lower risk for CAD compared to wild type carriers (0.64 95%CI 0.47–0.88; *p* = 0.007) (Table 2). However, the cardioprotective association of this variant could not be confirmed in Asian Indians in our study, despite with ~ 39% reduction in TG (Table 3). The reason might be the difference in MAF between Asian Indians (MAF = 0.00013) and Europeans (MAF = 0.002). Hence, a much larger sample would be required to confirm the cardioprotective role of this variant in other ethnic groups. With the exception of this splice variant, our data could not validate the cardioprotective role of other five rare functional variants (IVS1-2G-A (rs373975305); R19X (rs76353203); A43T (rs147210663); IVS3 + 1G-T (rs140621530); D65N (rs149707394); A10T (rs150821374). One of these six variants, A10T (rs150821374) was not found in any of our study cohorts. Generally, rare variants are population-specific and are often not seen in multiple ethnic populations [23, 41], but interestingly, these rare variants were present in Asian Indian Sikhs (Fig. 5). Nevertheless, their phenotypic effects were not replicated in these subjects as observed in earlier (predominantly European) studies [15, 18]. Our findings could not confirm the role of the most widely studied R19X (rs76353203) for its cardioprotective effect even in Europeans as first seen in Amish [15] and even in a Pakistani population [42]. Compared to the wild-type allele, 56 rare variant carriers of R19X (observed in the entire study) showed a 48.3% reduction in plasma TG (range 41.6–84.9 mg/dL), and a large majority were Europeans from UKBB. These results agree with earlier published findings of Crawford et al., [43] where known rare LoF variants did not reveal any protection from CAD in the European Americans from BioVU biobank.

Previous studies examining the function of ApoC-III in animal and cell culture models have shown that the increased expression of ApoC-III attenuates the activity of LPL and reduces the clearance of TG-rich lipoproteins [5, 44]. The increase in ApoC-III concentration in hepatocytes stimulates the assembly of TG-rich VLDL and inhibits VLDL lipolysis [5]. Both the increased expression of ApoC-III and hypertriglyceridemia (HTG) are predictors of CAD risk in diabetic patients [45]. Thus, it is possible that due to the abnormalities in ApoC-III, the same rare LoF or splice variant may not show similar phenotypic effects in multiple carriers due to pleiotropic effects of other genes within the LD region. Perhaps, because of these differences, we and other studies are unable to confirm that carriers of the LoF variants have low TG.

On the other hand, our data on 396,644 individuals using a common variant as a genetic instrument suggest that the *APOC3* elevates plasma TG levels through TG-raising mutations. However, a modest increase in the CAD risk of 3% despite a substantial increase of ~ 76% in TG levels by rs5128 suggests the possibility of pleiotropic effects of other variants or other nearby genes within the LD region of chromosome 11q23.

HTG is a multifactorial disease, and hence, the clinical management of these patients can be challenging. Adherence to lifestyle interventions such as weight loss, alcohol/tobacco withdrawal, use of lipid-lowering medications, and increased physical activity is emphasized as the essential principle for managing HTG in both the US and European guidelines [7]. Also, the development of novel technologies such as antisense oligonucleotides, siRNAs, dual apoC-II mimetic and apoC-III antagonist (called D6PV) offer promising results as potential therapies for HTG [6].

Substantial evidence in the literature on animal models and humans suggests that increased plasma ApoC-III levels increase with TG concentrations and are strong predictors of CAD [5, 44]. Although this study lacks measures on circulating levels of ApoC-III in all multi-ethnic datasets, plasma levels of ApoC-III in a small subset of Sikhs with wild type and mutant carriers of rs5128 revealed a 76% increase in TG levels and 37% increase in ApoC-III levels among mutant homozygous carriers compared to the wild type carriers. Of note, some wild-type allele carriers with very low TG (< 80 mg/dL) had high levels (>85th percentile) of ApoC-III (ranging between 24 and 29.5 mg/dL). From these results, it appears that other variants within *APOC3* could also be influencing the ApoC-III concentrations without affecting TG levels. Perhaps because of the missing information on ApoC-III levels, our MR results suggested only a modest increase in the risk for CAD. Availability of data with ApoC-III concentrations in the entire study subjects would be required to clarify the causal association of ApoC-III with CAD.

### Study strengths and limitations

The role of the common variants (identified in this study) for increasing serum TG has been reported in several small studies. Here, we have not only consolidated the multi-ethnic datasets and validated the associations of these common variants with TG and CAD, but also, for the first time are reporting the causal association of genetically raised TG with the risk for CAD using the MR approach using data from 396,644 participants from 10 independent multi-ethnic cohorts. Further, as earlier published studies have primarily examined individuals from European populations, we have included data from Asian Indians from India and other South Asians (UKBB), and Europeans and other ethnic groups to analyze the role of genetic variation in *APOC3* with CAD. For the first time, our study reports the role of earlier published rare variants for affecting TG and CAD in Asian Indians and other cohorts. Some limitations in this study deserve discussion. Firstly, even though our study included some independent cohorts of Asian Indians, the largest representation was still limited to the population of European origin. Thus, larger data sets of Asian Indians and other ethnic groups would be necessary to identify population-specific functional rare variants. In targeted sequencing of Punjabi Sikhs, we identified ~ 200 unique, rare variants; of these, 35 were associated with low TG levels (Supplementary Figure 1). However, their phenotypic effects could not be confirmed because of the small size of other Asian Indian cohorts and the lack of Sikh data. Secondly, the availability of ApoC-III concentrations in a large number of individuals would be required to clarify the causal association of ApoC-III with CAD. Thirdly, we evaluated the causal association with the CAD outcome using a single-sample MR and using the same datasets that could induce a bias if the genetic instrument is weak [46]. However, given our IV was strong and weighted on the TG effects, we do not expect that the data overlap had introduced any bias. Meta-analysis of 19 published studies, in fact, has reported a significant association between rs5128 and CAD in 11,186 subjects from multi-ethnic populations; however, because of the limited samples size of each study and heterogeneity, further validation was warranted [47]. Future independent evaluation using two-sample MR and using ApoC-III concentration in addition to TG would be helpful to confirm the causality. Fourthly, the data access was limited to summary statistics for many consortium cohorts that limited our ability to precisely correlate genotype-phenotype effects.

## Conclusions

Overall, these results highlight the challenges of inclusion of rare variant information for clinical risk assessment and generalizability of implementation of ApoC-III inhibition for treating atherosclerotic disease in dyslipidemia. On the other hand, our MR study suggests that the genetically regulated hypertriglyceridemic effects of ApoC-III may be partially associated with the increased risk for CAD, and other variants within *APOC3* and/or other nearby genes in the 11q23 cluster could be contributing to the increased ApoC-III levels and effecting CAD susceptibility. More studies on diverse populations would be needed to clarify the putative role of rare variants in *APOC3* or nearby genes (within the cluster) for their effects on dyslipidemia and CAD.

### Clinical perspective

Interindividual variation in circulating triglyceride (TG) is attributed to both genetic and environmental factors. Gene mapping studies have confirmed the strong influence of genetic factors for controlling circulating TG. However, the causal association of hypertriglyceridemia and the development of coronary artery disease (CAD) is unclear. Some loss-of-function rare variants in apolipoprotein-CIII *(APOC3)* have been suggested to decrease circulating TG and lower the risk for CAD. Yet, the results of relevant studies across diverse ethnic populations have been inconsistent and unclear. Population-based clinical and observational studies often suffer from confounding due to reverse causation because it is difficult to account for individual variation related to obesity, cultural and ethnic/genetic variations in such studies.

On the other hand, human genetic information used by Mendelian randomization (MR) methods up to some extent can outwit the noise of the reverse causation using suitable gene variants as a genetic instrument and may help assess the underlying mechanistic association between the disease phenotype and biomarker. Our MR study suggests that the genetically regulated hypertriglyceridemia via *APOC3* may be causally associated with the increased risk for CAD. From these findings, it appears that the siRNA/antisense inhibition of ApoC-III may be beneficial on the subsets of patients carrying these gain-of-function common variants (s).

## Supplementary Information

**Additional file 1: Supplementary Table 1.** Diagnostic criteria for CAD cases and non-CAD controls in participating study cohorts. **Supplementary Table 2.** Work performed at each study site. **Supplementary Table 3.** Meta-analysis results of the association of *APOC3* common variant rs5128 with plasma TG and the risk for CAD. **Supplementary Figure 1.** Detection of rare variants in *APOC3* gene region by targeted sequencing in Sikhs from AIDHS/SDS (Discovery). Dots in the graph represent variant (SNV). Figures on the x-axis denote the number of variants (SNVs), and the y-axis represents the corresponding mean plasma TG (mg/dL), and a cut-off of 100 mg/dL was used to define low or normal plasma TG levels. Of a total 201 rare variants or SNVs (MAF<1%) detected in Sikhs within *APOC3* region (116697024-116711387), only 35 (17%) had low TG (57-100 mg/dL) while a vast majority 166 (83%) of these were linked to high or very high TG (101-865 mg/dL).

## Data Availability

The datasets used and/or analyzed during the current study are available from the corresponding author on reasonable request.
