# Supplementary material for: APOC3 genetic variation, serum triglycerides, and risk of coronary artery disease in Asian Indians, Europeans, and other ethnic groups
Source: Lipids Health Dis. 2021 Sep 21;20:113. doi: 10.1186/s12944-021-01531-8 (PMC8456544; doi:10.1186/s12944-021-01531-8)
Supplement: Supplementary file 1 — Additional file 1: Supplementary Table 1. Diagnostic criteria for CAD cases and non-CAD controls in participating study cohorts. Supplementary Table 2. Work performed at each study site. Supplementary Table 3. Meta-analysis results of the association of APOC3 common variant rs5128 with plasma TG and the risk for CAD. Supplementary Figure 1. Detection of rare variants in APOC3 gene region by targeted sequencing in Sikhs from AIDHS/SDS (Discovery). Dots in the graph represent variant (SNV). Figures on the x-axis denote the number of variants (SNVs), and the y-axis represents the corresponding mean plasma TG (mg/dL), and a cut-off of 100 mg/dL was used to define low or normal plasma TG levels. Of a total 201 rare variants or SNVs (MAF<1%) detected in Sikhs within APOC3 region (116697024-116711387), only 35 (17%) had low TG (57-100 mg/dL) while a vast majority 166 (83%) of these were linked to high or very high TG (101-865 mg/dL). [file 12944_2021_1531_MOESM1_ESM.docx]

**Supplementary Materials**

***APOC3* genetic variation, serum triglycerides, and risk of coronary artery disease in Asian Indians, Europeans, and other ethnic groups**

Shiwali Goyal^1^, Yosuke Tanigawa^6^, Weihua Zhang^7,8^, Jin-Fang Chai^9^, Marcio Almeida^10^, Xueling Sim^9^, Megan Lerner^11^, Juliane Chainakul^12^, Jonathan Garcia Ramiu^12^, Chanel Seraphin^12^, Blair Apple^12^, April Vaughan^12^, James Muniu^1^, Juan Peralta^10^, Donna M Lehman^13^, Sarju Ralhan^14^, Gurpreet S Wander^14^, Jai Rup Singh^15^, Narinder K Mehra^16^, Evgeny Sidorov^12^, Marvin D Peyton^11^, Piers R Blackett^17^, Joanne E Curran^10^, E Shyong Tai^9,15,16^, Rob van Dam^8,18,20^, Ching-Yu Cheng^19,21,22^, Ravindranath Duggirala^10^, John Blangero^10^, John C Chambers^7,8,23,24,25^, Charumathi Sabanayagam^19,21^, Jaspal S Kooner^8,24,25,26^, Manuel A Rivas^6^, Christopher E Aston^1^, Dharambir K Sanghera^1,2,3,4,5^**^*^**

^1^Department of Pediatrics, College of Medicine, University of Oklahoma Health Sciences Center, Oklahoma City, OK 73104, USA

^2^Department of Pharmaceutical Sciences, University of Oklahoma Health Sciences Center, Oklahoma City, OK, USA

^3^Department of Physiology, College of Medicine, University of Oklahoma Health Sciences Center, Oklahoma City, OK, USA

^4^Oklahoma Center for Neuroscience, University of Oklahoma Health Sciences Center, Oklahoma City, OK, USA

^5^Harold Hamm Diabetes Center, University of Oklahoma Health Sciences Center, Oklahoma City, OK, USA

^6^Department of Biomedical Data Science, School of Medicine, Stanford University, CA, USA

^7^Department of Epidemiology and Biostatistics, Imperial College London, London W2 1PG, UK

^8^Department of Cardiology, Ealing Hospital, Middlesex UB1 3HW, UK

^9^Saw Swee Hock School of Public Health, National University of Singapore and National University Health System, Singapore

^10^Department of Human Genetics and South Texas Diabetes and Obesity Institute, University of Texas Rio Grande Valley, Brownsville, TX, USA

^11^Department of Surgery, Oklahoma University of health Sciences Center, Oklahoma City, OK, USA

^12^Department of Neurology, University of Oklahoma Health Sciences Center, 920 S.L Young Blvd #2040, 73104 Oklahoma City, OK, USA

^13^Departments of Medicine and Epidemiology and Biostatistics, University of Texas Health San Antonio, San Antonio, TX, USA

^14^Hero DMC Heart Institute, Ludhiana, Punjab, India

^15^Central University of Punjab, Bathinda, Punjab, India

^16^All India Institute of Medical Sciences and Research, New Delhi, India

^17^Department of Pediatrics, Section of Endocrinology, Oklahoma University of Health Sciences Center, Oklahoma City, OK, USA

^18^Department of Medicine, Yong Loo Lin School of Medicine, National University Health System, Singapore.

^19^Duke-NUS Medical School, Singapore

^20^Department of Nutrition, Harvard T.H. Chan School of Public Health, Boston, MA, USA.

^21^ Singapore Eye Research Institute, Singapore National Eye Centre, Singapore

^22^ National University of Singapore, Singapore

^23^Lee Kong Chan School of Medicine, Nanyang Technological University, Singapore 308232, Singapore

^24^Imperial College Healthcare NHS Trust, Imperial College London, London W12 0HS, UK

^25^MRC-PHE Centre for Environment and Health, Imperial College London, London W2 1PG, UK

^26^National Heart and Lung Institute, Imperial College London, London W12 0NN, UK

**Study cohorts**

**Asian Indian Diabetic Heart Study/Sikh Diabetes Study (AIDHS/SDS)**

A total of 4,659 individuals (age 20-93 years) were included in this study which included 820 participants for discovery and 3,839 participants for replication cohorts. These individuals were part of the Asian Indian Diabetic Heart Study (AIDHS)/Sikh Diabetes Study (SDS) recruited between 2002 to 2010 (1-4). All blood samples were obtained at the baseline visit. All patients participated in a comprehensive clinical assessment, family history, and baseline anthropometric and metabolic assessment at the time of recruitment as detailed in previous publications (1,3,4). All AIDHS/SDS protocols and consent documents were reviewed and approved by the University of Oklahoma Health Science Center's Institutional Review Board (IRB) as well as the Human Subject Protection (Ethics) committees at the participating hospitals and institutes in India (4-6).

***Sequencing cohort*:** Genomic DNA samples of 940 Sikh individuals including hypertriglyceridemia (HTG) cases (TG>150 mg/dL) and healthy controls with TG (<100 mg/dL) were sequenced with custom Nimblegen probes designed for targeted resequencing of 13 confirmed candidate genes for diabetic dyslipidemia in Sikhs as described previously.(6) HTG is broadly defined as fasting serum TG concentrations above the ninety-fifth percentile,(7) and was classified as mild HTG (150-399 mg/dL), high HTG (400-875 mg/dL), and severe HTG (>875 mg/dL). The non-HTG control participants were recruited from the same Punjabi Sikh community and from the same geographic location as the HTG participants as reported previously. (6) To reduce the confounding effects of *LPL* or other genes causing familial hyperchylomicronemia accompanied by extremely high serum TG, individuals with severe HTG (extremely high serum TG >1,000 mg/dL), those with familial chylomicronemia, hemochromatosis, or pancreatitis; those on lipid-lowering medication; and those with excessive alcohol intake (>400 mL/day) were excluded from the study.

**The London Life Sciences Prospective Population Study (LOLIPOP)**

LOLIPOP (8,9) is a prospective population study of ~28K Asian Indian and European White men and women, recruited at age 35-75 years from the lists of 58 General Practitioners in West London, UK, between May 1, 2002, and Sept 12, 2008. Asian Indians had all four grandparents born on the Indian subcontinent (India, Pakistan, Sri Lanka, or Bangladesh); Europeans were of self-reported white ancestry. At enrollment, all participants completed a structured assessment of cardiovascular and metabolic health, including anthropometry, medical history, and collection of blood samples for measurement of fasting glucose, lipid profile, and genotyping. Personal and family history were collected. Aliquots of whole blood were stored at -80°C for extraction of genomic DNA. The LOLIPOP study is approved by the National Research Ethics Service (07/H0712/150) and all participants gave written informed consent at enrollment.

**SINGAPORE**

**Multiethnic Cohort (MEC) (Asian Indians or Chinese)**

The Multi-Ethnic Cohort (MEC) (10) is a closed cohort that consists of Chinese, Malay, and Asian Indian participants, formed by combining two existing population-based studies conducted between 2004 to 2007, the Singapore Prospective Study Program (SP2) and the Singapore Cardiovascular Cohort Study (SCCS2). The MEC study was followed up with additional recruitment of participants between 2007 to 2010. A subset of Chinese and Indian participants who had whole-exome sequence data available were included in this analysis.

**Singapore Indian Eye Study (SINDI)**

The Singapore Indian Eye Study (SINDI) (11) is a population-based study that aims to document the prevalence, incidence, risk factors, and complications of eye diseases in Asian Indians, aged 40-79 years residing in the south-western part of Singapore. It is a part of the Singapore-Indian Chinese Cohort Eye Study (SICC).

**Singapore Diabetes Cohort Study and Singapore Prospective Study Program (DC-SP2)**

The Singapore Diabetes Cohort Study (DC) (12) (13,14) aims to examine genetic and environmental risk factors for diabetic complications. Since 2004, medical records and blood samples for >14,000 patients seen at polyclinics and hospitals were retrieved. The Singapore Prospective Study Program (SP2) consists of 6,968 participants from one of four previous cross-sectional studies: Thyroid and Heart Study 1982-1984, National Health Survey 1992, National University of Singapore Heart Study 1993-1995, or National Health Survey 1998. Each of these studies were sampled randomly from the Singapore population, aged 24 to 95 years, with disproportionate stratified sampling to increase the number of minority ethnic groups (Malays and Asian Indians). From 2003-2007, 5,157 participants were followed up for an interview and clinical examination.

**San Antonio Mexican American Family Studies (SAMAFS)**

The San Antonio Mexican American Family Study (SAMAFS) refers to the combination of two San Antonio-based family studies called the San Antonio Family Heart Study (SAFHS) (15) and the San Antonio Family Diabetes and Gallbladder Study (SAFDGS) (16). The SAFHS included 1,431 individuals in 42 large pedigrees at baseline. Probands were 40- to 60-years old, predominantly low-income Mexican Americans, selected at random without regard to presence or absence of disease, almost exclusively from Mexican American census tracts in San Antonio, TX. All 1^st^, 2^nd^, and 3^rd^-degree relatives of the proband and proband's spouse, aged ≥16 years, were eligible to participate. With additional recruitment, it was expanded to >1,900 individuals. The SAFDGS also began in 1991 like SAFHS, and originally included 579 examined individuals distributed across 32 large pedigrees. The sample was expanded to >900 individuals through additional recruitment and 8 new families were added. The probands for the SAFDGS were individuals with T2D. All 1^st^, 2^nd^, and 3^rd^-degree relatives, aged 18 or above, were invited to participate in the study. The SAMAFS participants have been followed in a mixed longitudinal fashion over a 25-year period, up to a maximum of 5 visits, and extensive phenotypic data, cross-sectional and longitudinal, are available. Phenotype data including traits such as BMI, lipids, blood pressure measures using standard protocols as described earlier. (15,17,18) The SAFHS and SAFDGS study-specific protocols have been approved by the Institutional Review Boards of the University of Texas Health San Antonio and the University of Texas Rio Grande Valley, and informed consent was obtained from all SAFHS and SAFDGS participants, respectively.

**Metabolome in Ischemic Stroke Study and Oklahoma Multiethnic CV Health Disparity Study (MISS-OLIVER)**

Metabolome in Acute Ischemic Stroke (MISS) (19) began in 2017, is a prospective study aimed to investigate ischemic stroke biomarker predictors by utilizing genomics, metabolomics, and Omics technologies. Patient recruitment is being carried out at the Comprehensive Stroke Center at the Oklahoma University Medical Center (OUMC). We are collecting serum and urine specimens during acute (<72 hours of stroke) and follow-up phases (within 3 months), and the recruitment is still ongoing. The goal is to compare profiles of stroke patients in acute, chronic stage with controls (without ischemic stroke). The OLIVER is a multiethnic population-based study that aims to investigate the disparity of cardiovascular disease health disparity in Oklahoma populations residing in rural and urban areas and patients from OUMC. Enrollment includes children (5 years and older) and adults and recording data of anthropometry, medical history, and collection of blood samples for measurement of fasting glucose, lipid profile, and genotyping. This investigation includes data of 512 subjects from the MISS-OLIVER study (218 US white; 294 US blacks and others) who were available with genome-wide genotypes.

**UK BIOBANK (UKBB)**

The data from UKBB (included in this study) comprise European white (EU) and people of Asian Indian descent based on self-reported ethnicity data and a subset of individuals confirmed using principal components. Ethics approval for the UKBB was obtained from the North West Centre for Research Ethics Committee (11/NW/0382). All the participants of UKBB provided written informed consent. Genotype data was comprised of a release version 2 for the directly genotyped variants, release version 3 for the imputed genotype dataset, and the initial 50k release of the exome sequencing dataset as described previously. (20,21) To minimize the variability due to structure in the dataset, analyses were restricted to unrelated individuals. Clinical characteristics of all study cohorts are summarized in **Table 1.** Diagnostic criteria for CAD cases and non-CAD controls in participating study cohorts are presented in **Supplementary Table 1**. Work performed at each study site is summarized in **Supplementary Table 2**.

**Targeted sequencing of lipid genes (AIDHS/SDS Discovery)**

Targeted sequencing was performed at the Northwest Genomics Center in the Department of Genome Sciences at the University of Washington through the RS&G Service sponsored by the National Heart Lung Blood Institute of the National Institutes of Health, as reported previously. (6) ***Library Production, Targeted Capture, Sequencing:*** Genomic DNA was extracted from whole blood or buffy coats using Qiagen kits (Qiagen, Chatsworth, CA, USA) or salting-out procedures described previously (10, 27). 1 µg of genomic DNA was sent to the Core lab at Northwest Genomics Center at the University of Washington for sequencing. The quality and integrity of DNA were checked at the Core lab using Agilent’s Analyzer and Tape Station reagents before target capture and library preparation. Library construction and custom capture have been automated (Perkin-Elmer Janus II) in a 96-well plate format. The purified DNA was subjected to a series of shotgun library construction steps, including fragmentation through acoustic sonication (Covaris), end-polishing and A-tailing, ligation of sequencing adaptors, and PCR amplification with 8 bp barcodes for multiplexing. Libraries undergo capture using the Roche/Nimblegen SeqCap EZ custom-designed probe. Prior to sequencing, the library concentration was determined by triplicate qPCR and molecular weight distributions verified on the Agilent Bioanalyzer (consistently 150 ± 15 bp). Barcoded libraries were pooled using liquid handling robotics prior to clustering (Illumina cBot) and loading. Massively parallel sequencing-by-synthesis with fluorescently labeled, reversibly terminating nucleotides was carried out on the HiSeq sequencer.

***Read Processing*:** Our sequencing pipeline is a combined suite of Illumina software and other “industry standard” software packages (i.e., Genome Analysis ToolKit [GATK], Picard, BWA, SAMTools, and in-house custom scripts) and consists of base calling, alignment, local realignment, duplicate removal, quality recalibration, data merging, variant detection, genotyping and annotation. The overall processing pipeline consists of the following elements: (1) base calls generated in real-time on the HiSeq2500 instrument (RTA 1.13.48.0) (2) demultiplexed, unaligned BAM files produced by Picard ExtractIlluminaBarcodes and IlluminaBasecallsToSam and (3) BAM files aligned to a human reference using BWA (Burrows-Wheeler Aligner; v0.6.2)[.](file:///C:\Users\dsangher\Documents\2021%20PROJECTS\APOCIII_2021\Final%20ApoCIII_jan%205\Edited%20APOC3%201-5-21\Edited%20APOC3%201-5-21\ONLINE%20SUPPLEMENTARY%20TEXT.docx#_ENREF_17) Read data from a flow cell lane is treated independently for alignment and QC purposes in instances where the merging of data from multiple lanes is required (e.g., for sample multiplexing). The samples were sequenced using paired-end 50bp reads and the insert sizes were at least 100 bp in length. Therefore, we expected to see ~150bp on the Bioanalyzer. Read-pairs not mapping within ± 2 standard deviations of the average library size (~150 ± 15 bp for the targeted region) were removed. All aligned read data are subject to the following steps: (1) “duplicate removal” was performed (i.e., the removal of reads with duplicate start positions; Picard MarkDuplicates; v1.70) (2) indel realignment was performed (GATK IndelRealigner; v1.6-11-g3b2fab9) resulting in improved base placement and lower false variant calls and (3) base qualities were recalibrated (GATK TableRecalibration; v1.6-11-g3b2fab9).

***Sequence Data Analysis QC*:** All sequence data underwent a QC protocol before they were released to the annotation group for further processing. This included an assessment of (1) total reads; (2) library complexity—the ratio of unique reads to total reads mapped to target. DNA libraries exhibiting low complexity are not cost-effective to finish; (3) capture efficiency—the ratio of reads mapped to human versus reads mapped to target; (4) coverage distribution—80% at 20X required for completion; (5) capture uniformity; (6) raw error rates; (7) Transition/Transversion ratio (Ti/Tv)-typically ~3 for known sites and ~2.5 for novel sites; (8) distribution of known and novel variants relative to dbSNP-typically <7% using dbSNP build 129 in samples of European ancestry; (9) fingerprint concordance >99%; (10) sample homozygosity and heterozygosity and (11) sample contamination validation. All QC metrics for both single-lane and merged data were reviewed by a sequence data analyst to identify data deviations from known or historical norms. Lanes/samples that failed QC were flagged in the system and could be re-queued for library prep (<5% failure) or further sequencing (<2% failure), depending upon the QC issue. Completion was defined as having >80% of the target at >20X coverage.

***Variant Detection*:** Variant detection and genotyping were performed using the UnifiedGenotyper (UG) tool from GATK (v1.6-11-g3b2fab9). Variant data for each sample were formatted (variant call format [VCF]) as “raw” calls that contain individual genotype data for one or multiple samples and flagged using the filtration walker (GATK) to mark sites that were of lower quality/false positives [e.g., low-quality scores (Q50), allelic imbalance (ABHet 0.75), long homopolymer runs (HRun> 3) and/or low quality by depth (QD < 5)].

***Variant Annotation:*** We used an automated pipeline for the annotation of variants derived from targeted sequencing data, the SeattleSeq Annotation Server (http://gvs.gs.washington.edu/ SeattleSeqAnnotation/). These publically accessible server returns annotations including dbSNP rsID (or whether the coding variant is novel), gene names and accession numbers, predicted functional effect (e.g., splice-site, nonsynonymous, missense, etc.), protein positions and amino-acid changes, PolyPhen predictions, conservation scores (e.g., PhastCons, GERP), ancestral allele, dbSNP allele frequencies, and known clinical associations. The annotation process has also been automated into our analysis pipeline to produce a standardized, formatted output (VCF-variant call format, described above).

Of the 940 sequenced samples, 820 passed the stringent quality control and were used for further analysis as described in detail previously.(6) All known rare variant carriers [rs373975305 (IVS1-2G-A); rs76353203 (R19X); rs138326449 (IVS2+1G-A); rs147210663 (A43T); rs140621530 (IVS3+1G-T)] observed in Sikhs were confirmed by Sanger sequencing.

**SNP Genotyping (AIDHS Replication)**

AIDHS/SDS replication cohorts were genotyped on Illumina platform using Human 660W Quad BeadChip panel; Illumina’s Global Screening Arrays with multi-disease content (GSA+); and GSA (Illumina, Inc., San Diego, CA) as explained in Saxena et al. (3,4). We performed pairwise identity-by-state clustering in PLINK across all individuals to assess population stratification; no population outliers were detected. Related individuals with pi-hat 0.3 and samples with, <93% call rate were excluded, as were SNPs with call rate <95%. Also excluded were SNPs with Hardy- Weinberg equilibrium (HWE) P <10^-6^ or minor allele frequency (MAF) <1% as described previously (4,22).

**Supplementary Table 1. Diagnostic criteria for CAD cases and non-CAD controls in participating study cohorts**

| **Cohort Name** | **Study type** | **Diagnostic criteria for CAD** | **Recruitment criteria for non-CAD controls** | **Reference** |
| --- | --- | --- | --- | --- |
| **AIDHS/SDS** | Case-control | Most of the CAD patients of AIDHS/SDS (~80%) had undergone coronary artery bypass graft (CABG) or angioplasty. In the rest of the patients, CAD was identified based on the medical records including the electrocardiographic (ECG) evidence of angina pain, coronary angiographic evidence of severe (greater than 50%) stenosis, or echocardiographic evidence of myocardial infarction | No history of thromboembolic disease or medication | (3) (22) |
| **LOLIPOP** | Prospective cohort | Myocardial infarction (chest pain associated with ECG evidence of myocardial infarction or raised cardiac enzymes or both); [unstable angina](https://www.sciencedirect.com/topics/medicine-and-dentistry/unstable-angina-pectoris) (cardiac pain associated with dynamic [ECG](https://www.sciencedirect.com/topics/medicine-and-dentistry/electrocardiogram) abnormalities); angiographically proven [coronary artery disease](https://www.sciencedirect.com/topics/medicine-and-dentistry/coronary-artery-disease) (>50% stenosis in one or more major epicardial vessel in multiple projections) | Participants free of heart disease or not on any medication | (23) |
| **SINGAPORE** | Longitudinal Study | Phenotype data on CAD was not available | - | - |
| **SAMAFS** | Family-based study | Prevalent cardiovascular disease was defined as self-reported physician-diagnosed stroke, heart attack, or heart surgery | Participants free of heart disease or not on any medication | (26) |
| **MISS_OLIVER** | Case-control | Patients that underwent coronary artery bypass graft (CABG) or angioplasty. CAD was also identified based on the medical records including the electrocardiographic (ECG) evidence of angina pain, coronary angiographic evidence of severe (greater than 50%) stenosis, or echocardiographic evidence of myocardial infarction | No history of thromboembolic disease or medication | (3) (19) |
| **UKBB** | Prospective study | HARD CAD was defined as fatal or non-fatal myocardial infarction, percutaneous transluminal coronary angioplasty (PTCA), or CABG. SOFT CAD includes all HARD CAD as well as chronic ischemic heart disease (IHD) and angina. All conditions were defined by either self-reported, hospital episode, or death registry data | Controls were defined as patients which were not a SOFT case after exclusions | (25) |

AIDHS/SDS: Asian Indian Diabetic Heart Study/Sikh Diabetes Study; LOLIPOP: The London Life Sciences Prospective Population study; SAMAFS: San Antonio Mexican American Family Studies; MISS_OLIVER: Metabolome in Ischemic Stroke Study and Oklahoma Multiethnic CV Health Disparity Study; UKBB: UK BIOBANK

**Supplementary Table 2. Work performed at each study site**

| **Cohort Name** | **Study type** | **Percentage of CAD cases** | **Work performed** | **Reference** |
| --- | --- | --- | --- | --- |
| AIDHS/SDS | Case-control | 17% | Discovery (Targeted sequencing)  Replication cohorts (GWAS) | (6) |
| LOLIPOP | Prospective cohort | Lolipop_AI=26%  Lollipop_EU=27% | LOLIPOP cohorts were genotyped on platforms including Illumina Human610 BeadChip (IA610 and EW610), Illumina HumanHap 300 BeadChip (IA317), Illumina HumanOmniExpressExome BeadChip (OmniEE), Illumina HumanOmniExpress BeadChip (OmniE_IA), Affymetrix 500K (EWA), and Perlegen custom (EWP and IAP). | (31, 32) |
| SINGAPORE | Longitudinal Study | NA | Exome sequencing was performed for individuals from the Multiethnic Cohort (MEC). DC-SP2 and SINDI were involved in the 13k exome sequence analysis. MEC was included in the subsequent 52k dataset. | (29, 30) |
| SAMAFS | Family-based study | ~18% | Whole-genome sequencing data was created on ~2400 samples of these for 586 individuals at >40x coverage was performed by T2D-Gene Consortium and remaining samples were sequenced using Illumina WGS technology at 30x coverage. | (27, 28) |
| MISS_OLIVER | Case-control | 27% | Genome-wide genotyping was performed on the Illumina platform using Illumina’s Global Screening Arrays with multi-disease content (GSA+); and GSA (Illumina, Inc., San Diego, CA). | (19) |
| UKBB | Prospective study | UKBB_AI=7%  UKBB_EU=4% | Three genetic datasets i.e. directly genotyped variants, imputed genotype dataset, and exome sequencing dataset in UK Biobank were used | (20, 21) |

AIDHS/SDS: Asian Indian Diabetic Heart Study/Sikh Diabetes Study; LOLIPOP: The London Life Sciences Prospective Population study; SAMAFS: San Antonio Mexican American Family Studies; MISS_OLIVER: Metabolome in Ischemic Stroke Study and Oklahoma Multiethnic CV Health Disparity Study; UKBB: UK BIOBANK

**Supplementary Table 3. Meta-analysis results of the association of *APOC3* common variant rs5128 with plasma TG and the risk for CAD**

| **Trait** | **SNP** | **Chromosome/**  **Position** | **Effect allele/**  **Other allele** | **EAF** | | **AIDHS/SDS**  **N=4,659** | **Meta-analysis**  **South Asians**  **N=26,596** | **Meta-analysis**  **Europeans**  **N=364,196** | **Meta-analysis**  **Others**  **N=5,852** | **Multiethnic**  **meta-analysis**  **N=396,644** |
| --- | --- | --- | --- | --- | --- | --- | --- | --- | --- | --- |
|  |  |  |  | **AIDHS & SA** | **EU** | **Beta (SE)**  **P-value** | **Beta (SE)**  **P-value** | **Beta (SE)**  **P-value** | **Beta (SE)**  **P-value** | **Beta (SE)**  **P-value** |
| **TG mg/dL** | **rs5128** | 11  116703640 | G/C | 0.33 | 0.11 | 0.06 (0.01)  7.18x10^-7^ | 0.09 (0.01)  8.10x10^-41^ | 0.19 (0.004)  2.36x10^-420^ | 0.09 (0.01)  1.15x10^-11^ | 0.15 (0.004)  2.80x10^-424^ |
|  |  |  |  |  |  | **OR (95% CI)**  **P-value** | **OR (95% CI)^*^**  **P-value** | **OR (95% CI)**  **P-value** | **OR (95% CI)^**^**  **P-value** | **OR (95% CI)**  **P-value** |
| **CAD** | **rs5128** | 11  116703640 | G/C | 0.33 | 0.11 | 1.01 (0.95-1.06)  0.813 | 1.01 (0.97-1.05)  0.610 | 1.03 (0.99-1.07)  0.151 | 1.13 (0.98-1.27)  0.119 | 1.03 (1.00-1.05)  0.042 |

TG: Triglycerides; CAD: Coronary Artery Disease; EAF: effect allele frequency; AIDHS: Asian Indian Diabetic Heart Study; SA: South Asians; EU: Europeans; Others include Mexican Americans, US Blacks, US Hispanics, Chinese from Singapore, and Multi-ethnic population from MISS_OLIVER; Metanalysis of South Asians include AIDHS/SDS, LOLIPOP Asian Indians, Singapore Asian Indians, and UK BioBank South Asians; Metanalysis of Europeans include LOLIPOP Europeans, and UK BioBank Europeans; Metanalysis of Others include Mexican Americans (SAMAFS), the multi-ethnic population of MISS_OLIVER and Singapore Chinese.

*Data from Singapore Asian Indians (MEC_ Indian and SINDI) and ** Data from Singapore Chinese (DC_SP2 and MEC_ Chinese) were not included because of the lack of information on CAD. For CAD multiethnic meta-analysis was performed in 390,932 individuals.

**Supplementary Figure 1.** **Detection of rare variants in *APOC3* gene region by targeted sequencing in Sikhs from AIDHS/SDS (Discovery).** Dots in the graph represent variant (SNV). Figures on the x-axis denote the number of variants (SNVs), and the y-axis represents the corresponding mean plasma TG (mg/dL), and a cut-off of 100 mg/dL was used to define low or normal plasma TG levels. Of a total 201 rare variants or SNVs (MAF<1%) detected in Sikhs within *APOC3* region (116697024-116711387), only 35 (17%) had low TG (57-100 mg/dL) while a vast majority 166 (83%) of these were linked to high or very high TG (101-865 mg/dL).

**References**

1. Sanghera DK, Bhatti JS, Bhatti GK et al. The Khatri Sikh Diabetes Study (SDS): Study design, methodology, sample collection, and initial results. *Hum Biol* 2006;78:43-63.

2. Sanghera DK, Nath SK, Ortega L et al. TCF7L2 polymorphisms are associated with type 2 diabetes in Khatri Sikhs from North India: Genetic variation affects lipid levels. *Ann Hum Genet* 2008;72:499-509.

3. Saxena R, Bjonnes A, Prescott J et al. Genome-wide association study identifies variants in casein kinase II (CSNK2A2) to be associated with leukocyte telomere length in a Punjabi Sikh diabetic cohort. *Circ Cardiovasc Genet* 2014;7:287-95.

4. Saxena R, Saleheen D, Been LF et al. Genome-Wide Association Study Identifies a Novel Locus Contributing to Type 2 Diabetes Susceptibility in Sikhs of Punjabi Origin From India. *Diabetes* 2013;62:1746-1755.

5. Sanghera DK, Ortega L, Han S et al. Impact of nine common type 2 diabetes risk polymorphisms in Asian Indian Sikhs: PPARG2 (Pro12Ala), IGF2BP2, TCF7L2 and FTO variants confer a significant risk. *BMC Medical Genetics* 2008;9:59.

6. Sanghera DK, Hopkins R, Malone-Perez MW et al. Targeted sequencing of candidate genes of dyslipidemia in Punjabi Sikhs: Population-specific rare variants in GCKR promote ectopic fat deposition. *PLoS One* 2019;14:e0211661.

7. Yuan G, Al-Shali KZ, Hegele RA. Hypertriglyceridemia: its etiology, effects and treatment. *CMAJ* 2007;176:1113-20.

8. Chambers JC, Zhang W, Sehmi J et al. Genome-wide association study identifies loci influencing concentrations of liver enzymes in plasma. *Nat Genet* 2011;43:1131-8.

9. Kooner JS, Chambers JC, Aguilar-Salinas CA et al. Genome-wide scan identifies variation in MLXIPL associated with plasma triglycerides. *Nat Genet* 2008;40:149-51.

10. Tan KHX, Tan LWL, Sim X et al. Cohort Profile: The Singapore Multi-Ethnic Cohort (MEC) study. *Int J Epidemiol* 2018;47:699-699j.

11. Lavanya R, Jeganathan VS, Zheng Y et al. Methodology of the Singapore Indian Chinese Cohort (SICC) eye study: quantifying ethnic variations in the epidemiology of eye diseases in Asians. *Ophthalmic Epidemiol* 2009;16:325-36.

12. Sim X, Ong RT, Suo C et al. Transferability of type 2 diabetes implicated loci in multi-ethnic cohorts from Southeast Asia. *PLoS Genet* 2011;7:e1001363.

13. Hughes K, Yeo PP, Lun KC et al. Cardiovascular diseases in Chinese, Malays, and Indians in Singapore. II. Differences in risk factor levels. *J Epidemiol Community Health* 1990;44:29-35.

14. Tan CE, Emmanuel SC, Tan BY, Jacob E. Prevalence of diabetes and ethnic differences in cardiovascular risk factors. The 1992 Singapore National Health Survey. *Diabetes Care* 1999;22:241-7.

15. Mitchell BD, Kammerer CM, Blangero J et al. Genetic and environmental contributions to cardiovascular risk factors in Mexican Americans. The San Antonio Family Heart Study. *Circulation* 1996;94:2159-70.

16. Hunt KJ, Lehman DM, Arya R et al. Genome-wide linkage analyses of type 2 diabetes in Mexican Americans: the San Antonio Family Diabetes/Gallbladder Study. *Diabetes* 2005;54:2655-62.

17. Duggirala R, Blangero J, Almasy L et al. Linkage of type 2 diabetes mellitus and of age at onset to a genetic location on chromosome 10q in Mexican Americans. *Am J Hum Genet* 1999;64:1127-40.

18. Melton PE, Carless MA, Curran JE et al. Genetic architecture of carotid artery intima-media thickness in Mexican Americans. *Circ Cardiovasc Genet* 2013;6:211-21.

19. Sidorov E, Bejar C, Xu C et al. Potential Metabolite Biomarkers for Acute Versus Chronic Stage of Ischemic Stroke: A Pilot Study. *J Stroke Cerebrovasc Dis* 2020;29:104618.

20. Bycroft C, Freeman C, Petkova D et al. The UK Biobank resource with deep phenotyping and genomic data. *Nature* 2018;562:203-209.

21. DeBoever C, Tanigawa Y, Aguirre M, McInnes G, Lavertu A, Rivas MA. Assessing Digital Phenotyping to Enhance Genetic Studies of Human Diseases. *Am J Hum Genet* 2020;106:611-622.

22. Sapkota BR, Hopkins R, Bjonnes A et al. Genome-wide association study of 25(OH) Vitamin D concentrations in Punjabi Sikhs: Results of the Asian Indian diabetic heart study. *J Steroid Biochem Mol Biol* 2016;158:149-56.

23. Chambers JC, Obeid OA, Refsum H, et al. Plasma homocysteine concentrations and risk of coronary heart disease in UK Indian Asian and European men. *Lancet*. 2000;355:523–27.

24. Mitchell, B.D., Kammerer, C.M., Blangero, J., et al. Genetic and environmental contributions to cardiovascular risk factors in Mexican Americans. The San Antonio Family Heart Study. *Circulation* 1996;94:2159–2170.

25. Nelson CP, Goel A, Butterworth AS, et al. Association analyses based on false discovery rate implicate new loci for coronary artery disease. *Nat Genet*. 2017 Sep;49(9):1385-1391. doi: 10.1038/ng.3913. Epub 2017 Jul 17. PMID: 28714975.

26. Hunt KJ, Diggirala R, Goring HH, et al. Genetic basis of variation in carotid artery plaque in the San Antonio Family Heart Study. Stroke 2002 Dec;33(12):2775-80. doi: 10.1161/01.str.0000043827.03966.ef. PMID: 12468769.

27. Jun G, Manning A, Almeida M, et al. Evaluating the contribution of rare variants to type 2 diabetes and related traits using pedigrees. Proc Natl Acad Sci U S A. 2018 Jan 9;115(2):379-384. doi: 10.1073/pnas.1705859115. Epub 2017 Dec 26. PMID: 29279374; PMCID: PMC5777025.

28. Blackburn NB, Michael LF, Meikle PJ, et al. Rare DEGS1 variant significantly alters de novo ceramide synthesis pathway. J Lipid Res. (2019). 60:1630-1639.

29. Flannick J, Mercader JM, Fuchsberger C, et al. Exome sequencing of 20,791 cases of type 2 diabetes and 24,440 controls. Nature. 2019 Jun;570(7759):71-76. doi: 10.1038/s41586-019-1231-2. Epub 2019 May 22. PMID: 31118516; PMCID: PMC6699738.

30. Flannick J, Fuchsberger C, Mahajan A, et al. Sequence data and association statistics from 12,940 type 2 diabetes cases and controls. Sci Data. 2017 Dec 19;4:170179. doi: 10.1038/sdata.2017.179. Erratum in: Sci Data. 2018 Jan 23;5:180002. PMID: 29257133; PMCID: PMC5735917.

31. Miller SA, Dykes DD, Polesky HF. A simple salting out procedure for extracting DNA from human nucleated cells. *Nucleic acids research* 1988; **16**(3): 1215.

32. Ng SB, Turner EH, Robertson PD, et al. Targeted capture and massively parallel sequencing of 12 human exomes. *Nature* 2009; **461**(7261): 272-6.
